# What Difficulties Does the Dentist Encounter from the Presence of Secondary Formations?

**Published:** 1896-05

**Authors:** E. F. Day


### What Difficulties Does the Dentist Encounter from the Presence of Secondary Formations?

BY E. F. DAY, L.D.S.

By the term "secondary formations" I have taken to mean:

1. Exostosis or enlargement of the cementum.

2. The formation of secondary dentine.

3. Calcareous degeneration of the pulp and formation of pulp stones.

The difficulties that a dentist would encounter from the presence of either of the above may be classified for the purpose of description under two heads, viz:

(a.) Difficulty of Diagnosis.

(b.) Difficulty in Treatment.

1. *Exostosis.*—The fact that we are unable to see the root of the tooth in this diseased condition, and that the symptoms are very vague, renders the diagnosis very troublesome. I am not aware of any diagnostic symptom which is characteristic of the disease or of any of the secondary formations, so that the inconvenience may vary from the dull, growing pain of an acute periostitic to the neuralgic pains following an exposed pulp.

Patients present themselves about the middle age, frequently with good sets of teeth, and complain of neuralgic pain around the teeth, face and head, which they can not locate. An examination will be made for caries, possibly there may be none. The question then arises, what is the cause of the neuralgia? And we must proceed to exclude all of the causes, such as a chill, the retarded eruption of third molars or any of the, may be, missing teeth, tumorous cysts, pregnancy and the like, but even by excluding these causes we cannot certainly say what the trouble is. Should, the patient be suffering from a rheumatic or gouty diathesis we may suspect exostosis. The treatment is almost as unsatisfactory as the foregoing diagnosis, and may be curative or radical. The radical treatment consists of extraction of the tooth or teeth, and in obstinate cases is the only course to insure cure. As curative measures, the application of counter irritants and the internal use of iodide of potassium is said to be good. As the deposit on the roots of teeth frequently enlarges the end of the root so much that it is larger than the crown, giving the tooth a dumbbell appearance, we find it very difficult to extract in these cases, and may have to resort to a large fissure by which we divide the alveolus after loosening up the tooth.

2. *Secondary Dentine.*—Since this secondary formation is of advantage to the individual as well as frequently necessary to the life of the pulp, we favor its progress as much as possible.

It sometimes happens that after a septum of secondary dentine has been formed the pulp dies, leaving this septum perfectly hard. The tooth presents itself for filling, and on excavating we find that there is no exposure of the pulp, and the walls are not softened above. In the earnest pursuit of our work we may have overlooked the fact that the tooth is not sensitive as many teeth we note, and we may have the mortification of filling over a dead pulp. This may not be a difficulty to all of us, but as I have had the misfortune to fill a tooth such as I have described, I thought it might be interesting to mention it. We all know that the application of a hot instrument or a piece of ice will clear up that difficulty.

3. And lastly, the calcareous degeneration of the pulp and formation of pulp stones.

When these conditions occur in old teeth it is a normal process, and since its diagnosis and treatment is of little value it requires no further comment.

But when pulp stones or nodules are formed in healthy teeth and at the same time we get an exposed pulp, and which we decide to kill in order to treat it, then the fun begins—I should say the difficulty begins.

We apply the arsenic and send the patient away for a day or two. Then we attempt to open up the pulp chamber (say, a molar) and after drilling for some time in that direction and no pulp chamber appearing (and as it was the first tooth of the kind I had treated) I began to think it was the queerest tooth I'd seen. On blowing the debris away I could see a small opening into the pulp chamber, or what was left of it, as I was now in the center of the tooth, and excepting this opening, all was calcified. I inserted a stiff bristle and by a lucky movement succeeded in dislodging the remaining part of the nodule which was blocking the way. Here I found the pulp so sensitive that I had to make another application of arsenic. On opening the tooth up again found other nodules in the canals. It seemed to be the delight of the pulp to get behind these nodules and defy my efforts to kill or extract it, simply asserting itself whenever my instrument (or hair tickler, as the patient called it) tried to grasp it. The tooth was eventually treated and fixed, in spite of the difficulties a dentist has to encounter due to the pressure of secondary formations.

